# Bacterial Agents and Their Multidrug‐Resistance Profile Among Patients Suspected of Bacterial Infections in Hakim Gizaw Hospital, Debre Berhan, Ethiopia: A Cross‐Sectional Study

**DOI:** 10.1002/hsr2.73346

**Published:** 2026-10-26

**Authors:** Tizazu Zenebe Zelelie, Ermias Endewunetu, Yosef Temesgen, Demissew Shenkute Gebreyes, Zenawork Sahile, Biniam Mekonnen, Getachew Bizuneh, Asrat Berihun, Berhanu Yitayew

**Affiliations:** ^1^ Department of Medical Laboratory Science, Asrat Woldeyes Health Science Campus Debre Berhan University Debre Berhan Ethiopia; ^2^ Department of Internal Medicine, Asrat Woldeyes Health Science Campus Debre Berhan University Debre Berhan Ethiopia; ^3^ Department of Surgery, Asrat Woldeyes Health Science Campus Debre Berhan University Debre Berhan Ethiopia; ^4^ Department of Paediatrics, Asrat Woldeyes Health Science Campus Debre Berhan University Debre Berhan Ethiopia; ^5^ Armauer Hansen Research Institute Addis Ababa Ethiopia

**Keywords:** antimicrobial resistance, bacteria, Ethiopia, multidrug resistant

## Abstract

**Background and Aims:**

Antimicrobial resistance is a global threat. Bacteria showed higher resistance in Ethiopia, and up‐to‐date data on it is urgently required for effective interventions. This study aimed to determine occurrence of multi‐drug‐resistant (MDR) bacterial isolates among patients presented to Hakim Gizaw Hospital (HGH).

**Methods:**

A health‐facility based cross sectional study was conducted in HGH, Debre Berhan, Ethiopia. All patients presented to HGH and diagnosed bacterial infections were eligible for the study. Depending on the clinical presentation of the patient, different clinical samples were investigated in microbiology laboratory following standard operating procedures. The analysis was carried out using SPSS version 27.

**Results:**

A total of 252 study participants were included in the analysis. The gender distribution was nearly equal, with 128 (50.8%, 128/252) females. Among all participants, 103 (40.9%, 103/252) were culture positive, irrespective of whether one or two bacterial isolates (polymicrobial) were identified. The prevalence of Gram‐positive and Gram‐negative bacteria was 26% (65/252) and 20% (50/252), respectively. Among all bacteria, *S. aureus* (40%, 46/115) was the predominant followed by *E. coli* (11.3%, 13/115). *E. coli* showed higher resistant to Ciprofloxacin (CIP), Doxycycline (DOX), Trimethoprim‐sulfamethozole (SXT), Cefoxitin (FOX), and Ceftaroline (CTR) (all ≥ 61.5%). *K. pneumonia* showed resistant from 27.3%–90.9%, with low resistant against GM and high resistant against Ampicillin (AM, 90.9%) and Amoxicilin‐clavulanate (AMC, 90.9%). *S. aureus* showed higher resistant against CIP (58.7%), SXT (60.9%), and Erythromycin (E, 67.4%). MDR rate was 66.1% (72/109). Among Gram‐negative bacteria, the prevalence of ESBLs and carbapenemase production was 66% (33/50) and 44% (22/50), respectively. Only age greater than 18 years old was significantly associated with MDR positive.

**Conclusions:**

Many bacterial infections were linked with high MDR bacterial isolates. However, few patient characteristics were shown statistical significance association. Thus, diagnosis and treatment of patients must be evidence based.

## 1 Introduction

Improvements in global health over recent decades are under threat due antimicrobial resistance (AMR) [1]. In some cases, pneumonia, other respiratory infections, and dysentery can no longer be cured with many older antimicrobials [1]. Currently, bacterial infections linked to one in eight global deaths [2]. Among total estimated 13.7 million infection related death in 2019, there were 7.7 million death associated with the 33 bacterial pathogens [3]. Five leading pathogens, *Staphylococcus aureus*, *Escherichia coli*, *Streptococcus pneumoniae*, *Klebsiella pneumoniae*, and *Pseudomonas aeruginosa* were responsible for 54.9% of deaths among the investigated bacteria [3].

Multi‐drug resistant (MDR) organisms of clinical concern include Methicillin‐resistant *Staphylococcus aureus*, Vancomycin‐resistant *Staphylococcus aureus*, Vancomycin‐resistant *Enterococci*, Extended spectrum ß‐lactamase (ESBLs)‐producing Gram‐negative bacilli, MDR *Streptococcus pneumoniae*, carbapenem‐resistant Enterobacteriaceae, MDR *Acinetobacter* and MDR *N. gonorrhea* [4, 5, 6]. In Gram‐negative bacteria, the resistance is mainly due to the rapid increase of ESBLs, and carbapenemase in *Klebsiella pneumoniae*, *Escherichia coli*, *Proteus mirabilis, Acinetobacter baumannii*, *Pseudomonas aeruginosa* and *Enterobacter spp*. and *Citrobacter spp* [7]. Globally there is serious and an urgent threats due to bacterial strains resistant to most antimicrobials including carbapenem and colistin, a last option drugs [8].

AMR is a growing challenge in Ethiopia and available reports indicate a trend towards increasing resistance rates among bacterial pathogens to commonly prescribed antimicrobials [9, 10, 11]. There is 63.8% of an overall prevalence of antimicrobial use, and most treatments are empiric (96.7%) in Ethiopia [12]. Inappropriate antimicrobial utilization and management is a problem in the country [13]. Infections with drug resistant organisms creates many health related problems such as improper antimicrobial agent prescription resulted in non‐beneficial effects in patients, high morbidity such as prolonged illness and hospitalization, and economic crisis due to other treatment options [14, 15]. In Ethiopia, mortality rate was associated with infections caused by ESBL‐producing Gram negative bacteria [16]. Data on the right potential pathogenic bacteria and its anti‐biogram profile could help to have the right treatment practices to reduce AMR, health costs, morbidity and mortality.

## 2 Methods

### 2.1 Study Design and Area

A hospital‐based cross‐sectional study was conducted in Hakim Gizaw Hospital (HGH), Debre Berhan, Ethiopia. The study was conducted from January to December 2024.

### 2.2 Study Population

All patients presenting HGH with clinical suspicion of bacterial infection were included in the study. Socio‐demographic and clinical data were collected using a structured questionnaire based on patient records and physician consultation. Both adult and pediatric patients suspected of bacterial infections were included, while those who had taken antimicrobials before culture were not indicated for culture testing, or declined participation were excluded.

### 2.3 Sample Size Determination

The sample size was calculated using a single population formula [17] based on a previous prevalence (*p* = 0.028), 95% confidence level, and margin of error (*d* = 0.06). The initial sample size was 215 and after adding a 10% non‐response rate, it became 237. However, a total of 252 patients were ultimately included in the study.

### 2.4 Data Collection Tool and Techniques

Socio‐demographic data, clinical presentations, laboratory results, and patient management data were collected using a structured questionnaire. Clinical and microbiological data were recorded, and clinical examinations were performed by clinicians or resident physicians during specimen collection in accordance with hospital protocols.

### 2.5 Specimen Type, Collection, Preparation, Transport and Storage

Samples based on the site of infection (blood, urine, stool, urethral discharge, swab from ulcer, venous blood and other body fluids) were collected following standard operating procedures (SOPs). Specimens were labeled with all the necessary information and transported using appropriate transport medium to microbiology laboratory.

### 2.6 Bacteria Isolation and Identification

Isolation and identification of bacteria were done based on standard microbiological procedure [18, 19]. The collected samples were first inoculated onto primary culture media based on the nutritional requirement of a particular pathogen according to SOPs. Isolation and identification were done to genus and species level using conventional microbiological techniques.

### 2.7 Antimicrobial Susceptibility Testing

Antimicrobial susceptibility tests (AST) for all isolates was done using the disc diffusion method (Kirby–Bauer method) according to Clinical and Laboratory Standards Institute guidelines (CLSI) [20]. A total of 16 antimicrobials were tested in this study. Briefly, Ciprofloxacin (CIP, 5 μg), Chloramphenicol (C, 30 μg), Gentamicin (GM, 10 μg), Doxycycline (DOX, 30 μg), Aztreonam (ATM, 30 μg), Trimethoprim‐sulfamethozole (SXT, 1.25/23.75 μg), Ampicillin (AM, 10μg), Amoxicilin‐clavulanate (AMC, 20/10 μg), Cefoxitin (FOX, 30 μg), Ceftaroline (CTR, 30 μg), Cefotaxime (CTX, 30 μg), Ceftazidime (CAZ, 30 μg), Cefepime (CPM, 30 μg), Meropenem (MEM, 10μg), Clindamycin (CM, 2 μg), and Erythromycin (E, 15 μg) included in the analysis. All the antimicrobial disks were from Oxoid (LTD, UK). *E. coli* ATCC (American Type Culture Collection) 25922 was used as a reference strain. ASTs were interpreted according to the CLSI guideline and categorized as susceptible (S), intermediate (I), or resistant (R). For the purposes of the present analysis, isolates categorized as intermediate or resistant were considered resistant. MDR was defined when isolate is resistant to at least one agent in three or more antimicrobial classes [21].

### 2.8 Phenotypic Detection of ESBL and Carbapenemase

Bacterial isolates resistant to cefotaxime, ceftazidime (30 µg), and meropenem in the screening test were subjected to the confirmatory testing. ESBL production was confirmed using the combination disk method (CDM), and carbapenemase production was confirmed using the modified disk method inactivation method (mCIM), following CLSI guidelines [20]. Briefly, the combination disk method was done on MHA by using ceftazidime (CAZ) and cefotaxime (CTX) alone and with ceftazidime + clavulanic acid (CAZ/CLA) and cefotaxime + clavulanic acid (CTX/CLA). The increase in zone size diameter by ≥ 5 mm for CTX/CLA and CAZ/CLA, when compared with that CTX and CAZ alone, was confirmed as the presence of ESBL. *K. pneumoniae* ATCC 700603 (ESBL positive) and *E. coli* ATCC 25922 (ESBL negative) were used for quality control. For mCIM, a bacterial suspension was incubated with a meropenem disc, which was then placed on muller Hinton agar inoculated with *E. coli* ATCC 29522. Briefly, a loopful (by using a 1 μL size loop) of the colony of the test isolates from overnight blood agar plate suspended in 2 mL of nutrient broth (Oxoid UK) and 10 μg of meropenem disk immersed in the nutrient broth (fully immersion). The tubes were incubated at 37°C in ambient air without agitation for 4 h ± 15 min. Subsequently, the meropenem disks were removed from the broth using an inoculation loop. Then, the disks were placed on Mueller‐Hinton agar plates freshly inoculated with a 0.5 McFarland suspension of *E. coli* ATCC 25922. Results were interpreted after incubation according to CLSI standards [20]. No or a small zone of inhibition on *E. coli* ATCC 25922 was considered confirmation that the suspected bacteria were carbapenemase producers.

### 2.9 Data Analysis

Data were analysed using SPSS (Statistical Package for Social Sciences) statistics version 27. Descriptive statistics (frequency and proportion) were computed. Associations between variables were assessed using the chi‐square test. Factors associated with MDR were identified using binary logistic regression [22]. Variables with *p* < 0.25 in univariate analysis and with previous knowledge were included in multivariate analysis. Statistical significance was considered as *p* < 0.05 (two‐sided). Model fitness was checked using the Hosmer‐Lemeshow test. Results were reported as odds ratios (OR) with 95% confidence intervals (CI).

### 2.10 Ethical Clearance

The research protocol was approved by Institutional review board of Astrat Woldeyes Health Science Campus, Debre Berhan University (Ref no: IRB 01/120/2016, with protocol number: IRB‐283 on April 18/2024). Written informed consent was obtained from each study participants following a clear explanation of study purpose.

## 3 Results

### 3.1 Socio‐Demographic Data

A total of 252 study participants (Supporting Information S1: Table S1 in Supplementary file) were included for the analysis. Majority (59.9%, 151/252) of the participants came from Debre Berhan, and others came from rural area of the town. The predominant study participants of this study were from the in‐patient department (67.1%, 169/252), and with age 18–64 years (57.1%, 144/252). The mean age of the study participants was 28.9 (with median, 28). Gender distribution of the study participants was almost equal for male (50.8%) and female. Detail socio‐demographic profile presented by Supporting Information S1: Table S1 in Supplementary file.

### 3.2 Clinical Characteristics of the Study Participants

The most common chief compliant among participants was dysuria (18%, 45/252) followed by abdominal pain (13.1%, 33/252), shortness of breath and fever (11%, 28/252 each), and swelling (9%, 23/252). Other symptoms were less frequent, while 23% (58/252) had varied complaints. Most participants presented with acute illness of 1‐7 days (52.4%, 132/252), and hospitalization history (24.2%, 61/252) in the last 90 days. The primary admission diagnosis was infectious diseases (46.4%, 117/252) (Supporting Information S1: Table S2 in Supplementary file).

### 3.3 Bacterial Isolates

Among 252 study participants, only 103 (40.9%, 103/252) were culture positive, with 91 clinical samples positive for single organism (36.1%, 91/252) and 12 clinical samples positive for two organisms (4.8%, 12/252). The103 patients yielded 115 isolates because 12 samples had polymicrobial growth (two bacteria). The prevalence of Gram‐positive bacteria (GPB) was 26% (65/252) with 57% (65/115) proportion with Gram negative bacteria (Figure 1). The predominant GPB were *S. aureus* (70.8%, 46/65), followed by Coagulase‐negative *Staphylococci* (CNS, 16.9%, 11/65), *S. agalactiae* (4.6%, 3/65), and other GPB (7.7%, 5/65) include *S. pneumonia* (*n* = 1), *S. pyogenes* (*n* = 1), *L. monocytogenes* (*n* = 1), and *C. diphtheriae* (*n* = 2). *E. coli* (26%, 13/50) was the leading Gram‐negative bacteria (GNB) in the present study. *E. aerogens* (24%, 12/50), *K. pneumonia* (22%, 11/50), *E. cloacae* (6%, 3/50), *Acinetobacter spp*. (6%, 3/50), other Enterobacterales (6%, 3/50), and other Gram negative (10%, 5/50) include *M. catarrhalis* (*n* = 1), *Neisseria species* (*n* = 3), and *P. aeruginosa* (*n* = 1) were found in this study (Figure 1).

**Figure 1:**
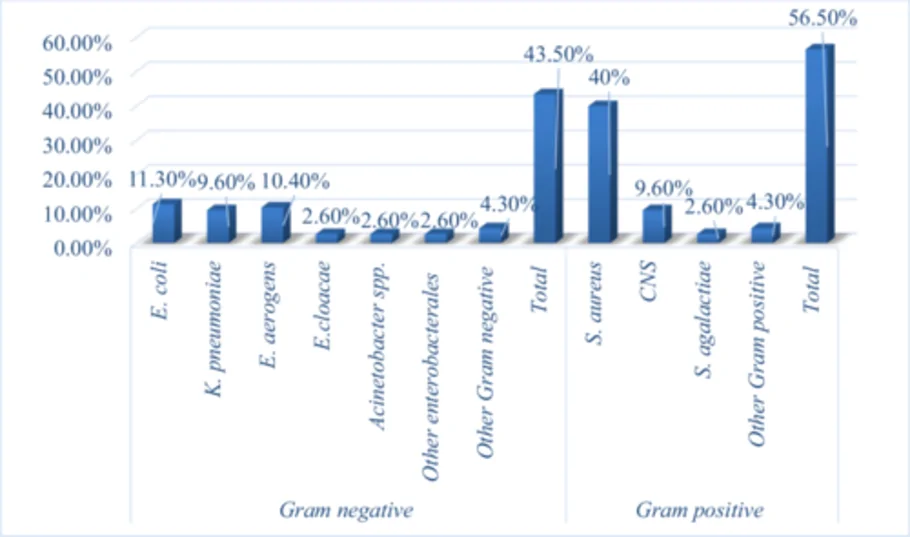
Types of bacterial isolates in the present study done in Debre Berhan Hakim Gizaw Hospital, 2024/2025. Other Enterobacterales = *C. freundii* (*n* = 1), *Enterobacter spp*.(*n* = 1), and *E. agglomerans* (*n* = 1); Other Gram negative bacteria = *M. catarrhalis* (*n *= 1), *Neisseria species* (*n* = 3), and *P. aeruginosa* (*n* = 1); Other Gram positive bacteria = *S. pneumonia* (*n* = 1), *S. pyogenes* (*n* = 1), *L. monocytogenes* (*n *= 1), and *C. diphtheriae* (*n* = 2).

### 3.4 Antimicrobial Resistance Profile

A total of 16 antimicrobials (14 for Gram‐negative and 9 for Gram‐positive bacteria) were included in the present antimicrobial susceptibility test (Supporting Information S1: Figure S1). GNB showed resistant from 0%–100% (total = 50) against different class of antimicrobials. *E. coli* showed higher resistant to CIP, DOX, SXT, FOX, and CTR (all ≥ 61.5%, from total = 13). *K. pneumonia* showed resistant from 27.3% to 90% (total = 11), with low resistant against GM and high resistant against AM (90.9%, 10/11) and AMC (90.9%, 10/11). *Enterobacter aerogenes*, *Enterobacter cloacae*, and other Enterobacterales were resistant to CIP, DOX, AM, AMC, FOX, CTR, CTX, CAZ, and CPM (63.6%‐100%). GPB also showed resistant from 0% to 100% (total=65). *S. aureus* showed higher resistant against CIP (58.7%, 27/46), SXT (60.9%, 28/46), and E (67.4%, 31/46). Lower resistant was seen by *S. aureus* against C (37.0%, 17/46), GM (17.4%, 8/46), and DOX (32.6%, 15/46). CNS showed relatively lower resistant (0%–27.2%) compared to *S. aureus*. *S. agalactiae* found resistant to C (33.3%, 1/3), E (66.7%, 2/3), and CM (33.3%, 1/3). Multiple bacterial isolates showed resistant to many antimicrobials (Supporting Information S1: Figure S1).

MDR rate was 66.1% (72/109) among the bacterial isolates subjected to antimicrobial susceptibility test in the present study (Table 1). Higher MDR rate was seen in GNB including *E. coli* (84.6%, 11/13), *K. pneumonia* (90.9%, 10/11), *E. aerogens* (91.7%, 11/12), other Enterobacterales (100%, 3/3). The MDR rate in Gram‐positive was 58.7% (27/46) by *S. aureus*, 33.3% (1/3) by *S. agalactiae*, and 27.3% (3/11) by CNS. A total of six *K. pneumonia* isolates (54.5%, 6/11) were resistant against more than eight different classes of antimicrobials. The prevalence of ESBLs in the present study was 66% (33/50) among Gram‐negative bacteria. Of the 33 ESBL‐producing isolates, 30.3% (10/33) were *E. aerogens*, 24.2% (8/33) were *E. coli* 24.2% (8/23) were *K. pneumoniae*, 9.1% (3/33) were *E. cloacae*, 9.1 (3/33) were other Enterobacterales, and 3.0% (1/33) were other GNB. A total of 22 bacterial isolates (22/50, 44%) were positive for carbapenemase production. The carbapenemase producers were *E. coli* (30.8%, 4/13), *K. pneumonia* (63.6%, 7/11), *E. aerogens* (58.3%, 7/12), other Gram‐negative bacteria (9.1%, 2/22), *E. cloacae* (4.5%, 1/22), and Other Enterobacterales (4.5%, 1/22).

**Table 1:** Antibiogram profile of bacterial isolates in the present study done in Debre Berhan Hakim Gizaw Hospital, 2024/2025.

Bacterial isolates	Level of antimicrobial resistance (%)									
	R0	R1	R2	R3	R4	R5	R6	R7	≥ R8	MDR
*E. coli* (*n* = 13)	0%	7.7%	7.7%	23.1%	15.4%	7.7%	23.1%	15.4%	0%	84.6%
*K. pneumonia* (*n* = 11)	0%	0%	9.1%	9.1%	18.2%	0%	9.1%	0%	54.5%	90.9%
*E. aerogens* (*n* = 12)	0%	8.3%	0%	8.3%	8.3%	16.7%	16.7%	16.7%	25%	91.7%
*E. cloacae* (*n* = 3)	0%	0%	0%	0%	66.7%	0%	33.3%	0%	0%	100%
Other Enterobacterales (*n* = 3)	0%	0%	0%	0%	33.3%	0%	33.3%	33.3%	0%	100%
*Acinetobacter spp*. (*n*= 3)	33.3%	0%	33.3%	0%	33.3%	0%	0%	0%	0%	33.3%
*S. aureus* (*n* = 46)	17.4%	15.2%	8.7%	6.5%	8.7%	4.3%	17.4%	17.4%	4.3%	58.7%
CNS (*n* = 11)	63.6%	9.1%	0%	9.1%	9.1%	0%	9.1%	0%	0%	27.3%
*S. agalactiae* (*n* = 3)	0%	0%	66.7%	33.3%	0%	0%	0%	0%	0%	33.3%
Others (*n* = 4)	50%	0%	0%	0%	25%	0%	0%	25%	0%	50%
Total (*n* = 109)	15.0%	9.3%	8.3%	9.3%	14.0%	4.7%	15.9%	12.1%	11.2%	66.1%

*Note:* R0: Sensitive to all antimicrobials tested (or stands for resistance for zero antimicrobial); R1, R2, R3, R4, R5, R6, R8: Resistant to one, two, three, four, five, six, eight and greater than eight antimicrobials, respectively. Other Enterobacterales = *C. freundii* (*n* = 1), *Enterobacter spp*. (*n* = 1), and *E. agglomerans* (*n* = 1). Others include *M. catarrhalis* (*n* = 1, and resistant to 7 antimicrobials), *S. pneumoniae* (*n* = 1, and resistant to four antimicrobials), *P. aeruginosa* (*n* = 1, and susceptible to all tested CLSI recommended antimicrobials), and *S. pyogenes* (*n* = 1, and susceptible to all tested CLSI recommended antimicrobials). *Note:* AST was not done for L. monocytogenes (*n* = 1), *C. diphtheriae* (*n* = 2) and *Neisseria species* (*n* = 3) from the present study.

Association between MDR positivity and under‐five age group, history of hospitalization, and antibiotic exposure in the last 90 days was statistically significant in the bivariate analysis (*p* < 0.05), indicating the presence of association (Supporting Information S1: Table S3 in Supplementary file). Similarly, antibiotic exposure in the last 90 days was significantly associated with ESBL‐producing bacteria and carbapenem resistant bacteria (*p* < 0.05). However, multivariate regression analysis showed a significant association only with age. Patients older than 18 years were nearly four times more likely to be MDR‐positive compared to under‐five children (95% CI: 3.053–41.285) (Table 2). Other socio‐demographic factors, including hospitalization history and antibiotic exposure in the last 90 days, did not show a statistically significant association with MDR positivity in adjusted analysis (Table 2). The Model fitness was checked and Hosmer‐Lemeshow test showed adequate goodness‐of‐fit of the final logistic regression model (χ^2^ = 15.04, df = 8, *p* = 0.058).

**Table 2:** The association between some socio‐demographic characteristics and multidrug resistant (MDR) positive.

Patient characteristics		MDR positive		Multivariate analysis	
		Yes	No	OR (95% CI)	*p* value
Patient address	Debre Berhan	44 (29.1%)	23 (15.2%)	1.00	
	Outside Debre Berhan	28 (27.7%)	8 (7.9%)	1.963 (0.631, 6.106)	> 0.05
Sex	Male	36 (28.1%)	15 (11.7%)	1.00	
	Female	36 (29%)	16 (12.9%)	0.461 (0.162, 1.311)	> 0.05
Age	< 5 years	27 (42.22%)	8 (12.5%)	1.00	
	5–18 years	2 (12.5%)	6 (37.5%)	0.635 (0.029, 14.052)	> 0.05
	> 18 years	43 (25%)	17 (9.9%)	3.780 (3.053, 41.285)	0.004
Educational level	Illiterate	12 (22.2%)	7 (13%)	1.00	
	Primary and junior	11 (28.2%)	5 (12.8%)	0.444 (0.046, 4.286)	> 0.05
	Preparatory	8 (27.6%)	2 (6.9%)	0.120 (0.009, 1.619)	> 0.05
	College or University	9 (17%)	6 (11.3%)	0.202 (0.016, 2.626)	> 0.05
	Others^¥^	32 (41.6%)	11 (14.3%)	0.790 (0.068, 9.165)	> 0.05
Occupation	Unemployed	11 (42.3%)	3 (11.5%)		
	Private employed	4 (12.5%)	2 (6.2%)	0.525 (0.037, 7.480)	> 0.05
	Government employed	12 (30%)	6 (15%)	2.659 (0.111, 63.888)	> 0.05
	Self employed	5 (20%)	3 (12%)	1.057 (0.056, 19.906)	> 0.05
	Others^£^	12 (21.1%)	7 (12.3%)	3.395 (0.173, 66.503)	> 0.05
	Not applicable	28 (38.9%)	10 (13.9%)	1.426 (0.096, 21.096)	> 0.05
Family income (monthly in birr)	< 5000	49 (27.7%)	20 (11.3%)	1.00	
	5000–10,000	16 (25.8%)	10 (16.1%)	2.786 (0.292, 26.552)	> 0.05
	> 10000	7 (53.8%)	1 (7.7%)	4.217 (0.374, 47.531)	> 0.05
Duration of illness	< 7 days	56 (28.7%)	24 (12.3%)	1.00	
	7–28 days	12 (31.6%)	4 (10.5%)	0.341 (0.053, 2.208)	> 0.05
	> 28 days	4 (21.1%)	3 (15.8%)	0.281 (0.030, 2.600)	> 0.05
Hospitalization history	Yes	25 (41%)	7 (11.55)	1.00	
	No	47 (24.6%)	24 (12.6%)	0.485 (0.136, 1.730)	> 0.05
Surgery	Yes	11 (22.9%)	7 (14.6%)	1.00	
	No	61 (29.9%)	24 (11.8%)	1.666 (0.378, 7.344)	> 0.05
Comorbidity	Yes	21 (27.3%)	9 (11.7%)	1.00	
	No	51 (29.1%)	22 (12.6%)	1.632 (0.514, 5.186)	> 0.05
Antibiotic exposure in the last 90 days	Yes	35 (40.7%)	10 (11.6%)	1.00	
	No	37 (22.3%)	21 (12.7%)	0.702 (0.211, 2.341)	> 0.05

*Note:* ¥ Other Include study participants not eligible to education (less than 7 years); £ other Include retired, farming, doing paid work, and non‐regular businesses.

Abbreviations: CI, confidence interval; MDR, multi‐drug resistance; OR, odd ratio.

## 4 Discussions

In this study, culture positivity was nearly 41%. GPB predominated over GNB. In 7 of 12 sample types, GPB were significantly more common (*p* < 0.05), likely contributing to their overall predominance. Consistent with the present findings, Alemnew et al. [23] reported 15.5% GPB and 10.5% GNB isolates in Ethiopia. Similar trends were observed in India (54.2% GPB vs. 24.9% GNB) [24] and China (70.7% GPB vs. 29.3% GNB [25]. However, many other studies reported inconsistent finding with the present study. Among these studies, 32.2% Gram‐positive and 67.8% GNB [26], and 16.4% GPB and 53.6% GNB [27] has been reported in Ethiopia. In addition, 16% GPB and 84% GNB in Tanzania [28], and 31.1% GPB and 68.9% GNB in Bangladesh [29] were reported. Detection of bacterial isolates may vary due to factors such as sample type, prior antibiotic use, pathogen characteristics, sample collection and handling, and time to positivity [30]. The discrepancy between the present study and others may be due to difference in types of sample proportion and the nature of the study design. Blood culture studies in Ethiopia [31] and China [25] similarly reported higher proportions of GPB compared to GNB. The predominance of blood samples in this study may have contributed to the higher isolation of GPB. Overall, the findings highlight the clinical significance of GPB pathogens among patients presenting to the hospital.

The predominate GPB were *S. aureus* (~ 71%), Coagulase‐negative *staphylococci* (CNS) (~ 17%), and *S. agalactiae* (4.6%), consistent with finding from Ethiopia [32]. *S. aureus* was the main isolate from abscess and wound samples, likely due to its role as a common skin colonizer that exploits disrupted barriers. It is also a leading cause of bacteraemia, endocarditis, and other invasive infections [33]. CNS, more frequently in urethral discharge, are important hospital pathogens, especially in patients with weakened immunity or indwelling devices [34]. *S. agalactiae* is a key cause of neonatal sepsis and meningitis, and is also linked to genitourinary and invasive infections in adults [35]. Other GPB (7.7%) including *S. pneumonia*, *S. pyogenes*, *L. monocytogenes*, and *C. diphtheriae* were also identified. The present findings highlight that GPB are playing a significant role in bacterial infections and call an urgent intervention including improved hand hygiene.

The predominant GNB were *E. coli* (26%), *E. aerogenes* (24%), and *K. pneumoniae* (22%), consistent with previous studies [36, 37]. Other isolates included *E. cloacae* and *Acinetobacter spp*. (6% each), along with additional Enterobacterales and non‐fermenters. MDR GNB are major causes of hospital‐acquired infections and poses serious treatment challenges due to diverse resistance mechanisms [38]. The finding highlights the need for continuous monitoring of GNB pathogens at the hospital level.

In this study, bacterial isolates showed varied resistance (0‐100%) to different antimicrobials. Among GNB, *E. coli* showed high resistance to CIP, DOX, SXT, FOX, and CTR (≥ 61.5%), consistent with previous studies in Ethiopia [26, 37, 39] and abroad, including Gambia [40], Sudan [41], and Bangladesh [42]. As a prevalent bacterium in different interfaces (human, animal, and environment), *E. coli* serves as a key AMR indicator, and its resistance patterns reflect broader trends in GNB [43]. *K. pneumoniae* showed nearly 64%‐91% resistance to multiple drugs, higher than reports from Gambia [40], likely due to differences in study design and timing. *E. aerogenes*, *E. cloacae*, and other Enterobacterales also showed MDR, highlighting their role as opportunistic pathogens with high potential for horizontal gene transfer [44]. *Enterobacter* is a versatile bacterium able to promptly respond to the antibiotic treatment in the colonized patient and has ability to horizontal transmission of mobile elements containing antibiotic resistance genes [44]. The finding reflects the growing AMR challenge in the study area and align with evidence from sub‐Saharan Africa, where resistance among Enterobacterales is particularly severe in Eastern Africa, including Ethiopia [45].

Among GPB, *S. aureus* showed high resistance to E (67.4%), SXT (~ 61%), FOX and CTR (39.1%), and C (37%), similar to findings by Obakiro et al [39]. *S. aureus* poses a global threat, especially in healthcare settings, and classified as a high priority pathogen in the 2024 WHO bacterial priority pathogen list (BPPL) [46]. *S. agalactiae* and *S. pneumoniae* also showed resistance to commonly used antimicrobials, with both included in the medium priority category of the WHO BPPL, highlighting their public health importance [46]. MDR GPB infections increase morbidity, mortality and healthcare costs [1, 46, 47]. The finding underscores the need for diagnostic‐guided treatment and implementation of antimicrobial stewardship (ASP) programs to optimize patient management.

The present study found a high MDR rate (66.1%), consistent with previous reports in Ethiopia (61.8%–88.8%) [9, 26, 48] and other countries such as Bangladesh (83.4%), Egypt (52.4%), and Tanzania (47.3%) [28, 29, 49]. MDR was particularly high among *E. cloacae* (100%), *E. aerogenes* (91.7%), *K. pneumoniae* (90.9%), *E. coli* (84.6), and *S. suraus* (58.7%), reflecting trends reported elsewhere [48]. Age‐specific patterns showed varied MDR. MDR prevalence was highest among adults (> 18 years), unlike some studies reporting higher rates in elderly or pediatric populations [50, 51]. This inconsistency across studies may be attributed to several factors including difference in clinical conditions (e.g. UTI vas BSI), variation in the specific bacterial species, population characteristics (age, comorbidity, prior antibiotic exposure), and geographical variations (like prescription practice, IPC measures, and local resistance ecology). The finding highlights the urgent need for ASP programs to improve patient management and curb further MDR emergence.

The rise in MDR infections is largely driven by the lack of ASP and irrational antimicrobial use in hospital [38]. Implementing effective ASP can prevent the spread of MDR bacteria [52]. The present study provides evidence to support evidence‐based stewardship policies and timely antibiogram development, which help guide therapy, detect outbreaks, and inform antimicrobial policy [40]. Understanding the drivers of MDR need further analysis and urgent implementation of stewardship programs is warranted.

The prevalence of ESBL‐producing GNB in this study was 66% (33/50), consistent with reports from Ethiopia (64.8%‐66.6%) [37, 53]. The main ESBL producers were *E. aerogenes* (30.3%), *E. coli* (24.2%), *K. pneumoniae* (24.2%), and *E. cloacae* (9.1%). Third‐generation cephalosporin‐resistant Enterobacterales are classified as critical priority pathogens by WHO 2024 BPPL, highlighting their public importance [46]. Carbapenem‐resistant GNB were observed in 44% (22/50) of the isolates, mainly *K. pneumoniae* (31.8%), and *E. aerogens* (31.8%), and *E. coli* (18.2%), indicating that carbapenem resistance is spreading beyond *K. pneumoniae* [38]. The finding underscores the threats to empiric therapy [38], the need for routine detection of ESBLs and carabapenemases, and the importance of ASP and infection control to improve outcomes in critically ill patients.

The present findings provide important evidence on bacterial isolates, highlighting the magnitude of the problem and signaling the need for urgent public health action. These data are valuable for guiding empirical treatment based on local antibiograms. The study shows a significant burden of AMR, underscoring the importance of routine antimicrobial susceptibility tests to inform effective interventions and promote rational antibiotic use. Additionally, the findings emphasize the need to strongly implement ASP in the hospital. ASP are a key component of national AMR strategies and have shown to reduce AMR [54, 55]. Thus, evidence‐based approaches, including culture and susceptibility testing, should guide patient management with implementation of ASP and infection prevention measures.

## Author Contributions

**Tizazu Zenebe:** conceptualization, methodology, project administration, writing – original draft, writing – review and editing, supervision. **Ermias Endewunetu, Yosef Temesgen, and Demssew Shenkute:** methodology, software, data curation, formal analysis, writing – review and editing, visualization, validation. **Zenawork Sahile, Biniam Mekonnen, and Getachew Bizuneh:** conceptualization, funding acquisition, investigation, writing – original draft, writing – review and editing. **Asrat Berihun and Berhanu Yitayew:** writing – original draft, writing – review and editing. All authors have read and approved the final version of the manuscript.

## Conflicts of Interest

The authors declare no conflicts of interest.

## Transparency Statement

The lead author, Tizazu Zenebe, affirms that this manuscript is an honest, accurate, and transparent account of the study being reported; that no important aspects of the study have been omitted; and that any discrepancies from the study as planned (and, if relevant, registered) have been explained.

## Supporting information


Supporting File

## Data Availability

The data that support the findings of this study are available from the corresponding author upon reasonable request.
