# Supplementary material for: Bacterial Agents and Their Multidrug‐Resistance Profile Among Patients Suspected of Bacterial Infections in Hakim Gizaw Hospital, Debre Berhan, Ethiopia: A Cross‐Sectional Study
Source: Health Sci Rep. 2026 Oct 26;9(11):e73346. doi: 10.1002/hsr2.73346 (PMC13697516; doi:10.1002/hsr2.73346)
Supplement: Supplementary file 1 [file HSR2-9-e73346-s001.docx]

|  | E. coli (n=13) | K. pneumonia (n=11) | E. aerogens (n=12) | E. cloacae (n=3) | Other enterobacterales (n=3) | M. catarrhalis (n=1) | P. aeruginosa(n=1) | Acinetobacter spp. (n=3) | S. aureus (n=46) | CNS (n=11) | S. agalactiae (n=3) | S. pneumoniae (n=1) | S. pyogenes (n=1) |
| --- | --- | --- | --- | --- | --- | --- | --- | --- | --- | --- | --- | --- | --- |
| CIP | 61.5 | 72.7 | 75 | 66.7 | 100 | 100 | 0 | 66.7 | 58.7 | 27.2 | 0 | 0 | 0 |
| C | 46.2 | 72.7 | 50 | 33.3 | 66.7 | 100 | 0 | 0 | 37 | 18.1 | 33.3 | 0 | 0 |
| GM | 0 | 27.3 | 25 | 33.3 | 33.3 | 100 | 0 | 0 | 17.4 | 0 | 0 | 0 | 0 |
| DOX | 61.5 | 63.6 | 75 | 100 | 66.7 | 100 | 0 | 0 | 32.6 | 18.1 | 0 | 100 | 0 |
| ATM | 7.7 | 63.6 | 41.7 | 0 | 0 | 0 | 0 | 0 | 0 | 0 | 0 | 0 | 0 |
| SXT | 61.5 | 72.7 | 66.7 | 0 | 100 | 100 | 0 | 33.3 | 60.9 | 18.1 | 0 | 0 | 0 |
| AM | 92.3 | 90.9 | 100 | 100 | 100 | 100 | 0 | 0 | 0 | 0 | 0 | 0 | 0 |
| AMC | 92.3 | 90.9 | 91.7 | 100 | 100 | 100 | 0 | 0 | 0 | 0 | 0 | 0 | 0 |
| FOX | 61.5 | 72.7 | 83.3 | 100 | 100 | 100 | 0 | 0 | 39.1 | 27.2 | 0 | 0 | 0 |
| CTR | 61.5 | 63.6 | 66.7 | 100 | 100 | 0 | 0 | 0 | 39.1 | 18.1 | 0 | 0 | 0 |
| CTX | 61.5 | 72.7 | 83.3 | 100 | 100 | 0 | 0 | 33.3 | 0 | 0 | 0 | 0 | 0 |
| CAZ | 61.5 | 72.7 | 83.3 | 100 | 100 | 0 | 0 | 33.3 | 0 | 0 | 0 | 0 | 0 |
| CPM | 53.9 | 81.8 | 75 | 0 | 100 | 0 | 0 | 33.3 | 0 | 0 | 0 | 0 | 0 |
| MEM | 30.8 | 63.6 | 58.3 | 0 | 33.3 | 0 | 0 | 33.3 | 0 | 0 | 0 | 0 | 0 |
| E | 0 | 0 | 0 | 0 | 0 | 0 | 0 | 0 | 67.4 | 18.1 | 66.7 | 100 | 0 |
| CM | 0 | 0 | 0 | 0 | 0 | 0 | 0 | 0 | 45.7 | 9.1 | 33.3 | 100 | 0 |

Figure S1. Antimicrobial resistance profile of bacteria against different class of antimicrobials in the present study done in Debre Berhan Hakim Gizaw Hospital, 2024/2025. Abbreviations: Ciprofloxacin=CIP, Chloramphenicol=C, Gentamicin =GM, Doxycycline=DOX, Aztreonam=ATM, Trimethoprim-sulfamethozole=SXT, Ampicillin=AM, Amoxicilin-clavulanate=AMC, Cefoxitin=FOX, Ceftaroline=CTR, Cefotaxime=CTX, Ceftazidime=CAZ, Cefepime =CPM, , Meropenem=MEM; Clindamycin=CM, Erythromycin=E; Other enterobacterales = *C. freundii (n=1), Enterobacter spp.(n=1),* and *E .agglomerans(n=1); AST was not done for L. monocytogenes(n=1), C.diphtheriae (n=2) and* Neisseria species (n=3). Each colour represents the number of bacteria resistant to the specific antimicrobials.

Table S1. Socio-demographic profile of the patients participated in the study done in Debre Berhan Hakim Gizaw Hospital, 2024/2025.

| **Characteristics** | | **Frequency** | **Percent (%)** |
| --- | --- | --- | --- |
| Patient address | Debre Berhan | 151 | 59.9 |
|  | Outside Debre Berhan | 101 | 40.1 |
| Resident of the patients | Rural | 155 | 61.5 |
|  | Town | 97 | 38.5 |
| Types of departments | Out-patient department | 55 | 21.8 |
|  | In-patient department | 169 | 67.1 |
|  | Emergency department | 28 | 11.1 |
| Age of the patients | <5 years | 64 | 25.4 |
|  | 5-18 years | 16 | 6.3 |
|  | >18 years | 172 | 68.3 |
| Sex of the patients | Male | 128 | 50.8 |
|  | Female | 124 | 49.2 |
| Educational level of the patients | Illiterate | 54 | 21.4 |
|  | Primary and junior | 39 | 15.5 |
|  | Preparatory | 29 | 11.5 |
|  | College or University | 53 | 21.0 |
|  | Others^*^ | 77 | 30.6 |
| Marital status of the patients | Single | 48 | 19.0 |
|  | Married | 123 | 48.8 |
|  | Other | 8 | 3.2 |
|  | Not applicable | 73 | 29.0 |
| Occupational status | Unemployed | 26 | 10.3 |
|  | Private employed | 32 | 12.7 |
|  | Government employed | 40 | 15.9 |
|  | Self employed | 25 | 9.9 |
|  | Others^θ^ | 57 | 22.6 |
|  | Not applicable | 72 | 28.6 |

**Notes:** * Include study participants not eligible to education (less than 7 years); θ Include retired, farming, doing paid work, and non-regular businesses.

Table S2. Patient clinical characteristics of the study participants of this study conducted in, Hakim Gizaw Hospital, Debre Berhan, 2024.

| Characteristics | | Frequency | Percent (%) |
| --- | --- | --- | --- |
| Acute illness duration | <1 day | 63 | 25.0 |
|  | 1-7 days | 132 | 52.4 |
|  | 8-14 days | 31 | 12.3 |
|  | 15-30 days | 18 | 7.1 |
|  | >30 days | 8 | 3.2 |
| Hospitalization history | Yes | 61 | 24.2 |
|  | No | 191 | 75.8 |
| Health care visit history | Yes | 120 | 47.6 |
|  | No | 132 | 52.4 |
| Primary admission diagnosis | Orthopaedic conditions | 11 | 4.4 |
|  | Cardiovascular conditions | 19 | 7.5 |
|  | Neurological conditions | 12 | 4.8 |
|  | Infectious disease | 117 | 46.4 |
|  | Gastrointestinal disorder | 32 | 12.7 |
|  | Others^*^ | 18 | 7.1 |
|  | Not admitted | 43 | 17.1 |
| Surgery | Yes | 48 | 19.0 |
|  | No | 204 | 81.0 |
| Comorbidity | Yes | 77 | 30.6 |
|  | No | 175 | 69.4 |

**Note:** *Others include Endocrine/metabolic disorder, genitourinary disorder, respiratory disorder, prematurity, and malnutrition

**Table S3. Some socio-demographic and clinical characteristics of the study participants in relation positive to MDR, ESBL and carbapenemase**

| **Patient characteristics** | | **MDR positive** | | | **ESBL positive** | | | **Carbapenemase positive** | | |
| --- | --- | --- | --- | --- | --- | --- | --- | --- | --- | --- |
|  |  | **Yes** | **No** | **P value** | **Yes** | **No** | **P value** | **Yes** | **No** | **P value** |
| Patient address | Debre Berhan | 44 (29.1%) | 23 (15.2%) | > 0.05 | 24 (15.9%) | 6 (4%) | > 0.05 | 14 (9.3%) | 16 (10.6%) | > 0.05 |
|  | Outside Debre Berhan | 28 (27.7%) | 8 (7.9%) |  | 12 (11.9%) | 1 (1%) |  | 9 (8.9%) | 4 (4%) |  |
| Sex | Male | 36 (28.1%) | 15 (11.7%) | > 0.05 | 16 (12.5%) | 3 (2.3%) | > 0.05 | 7 (5.5%) | 12 (9.4%) | > 0.05 |
|  | Female | 36 (29%) | 16 (12.9%) |  | 20 (16.1%) | 4 (3.2%) |  | 16 (12.9%) | 8 (6.5%) |  |
| Age | <5 years | 27 (42.2%) | 8 (12.5%) | 0.001 | 13 (20.3%) | 2 (3.1%) | > 0.05 | 11 (17.2%) | 4 (6.2%) | > 0.05 |
|  | 5-18 years | 2 (12.5%) | 6 (37.5%) |  | 1 (6.2%) | 1 (6.2%) |  | 1 (6.2%) | 1 (6.2%) |  |
|  | >18 years | 43 (25%) | 17 (9.9%) |  | 22 (12.8%) | 4 (2.3%) |  | 11 (6.4%) | 15 (8.7%) |  |
| Educational level | Illiterate | 12 (22.2%) | 7 (13%) | > 0.05 | 8 (14.8%) | 1 (1.9%) | > 0.05 | 4 (7.4%) | 5 (9.3%) | > 0.05 |
|  | Primary and junior | 11 (28.2%) | 5 (12.8%) |  | 6 (15.4%) | 1 (2.6%) |  | 5 (12.8%) | 2 (5.1%) |  |
|  | Preparatory | 8 (27.6%) | 2 (6.9%) |  | 5 (17.2%) | 1 (3.4%) |  | 1 (3.4%) | 5 (17.2%) |  |
|  | College or University | 9 (17%) | 6 (11.3%) |  | 3 (5.7%) | 2 (3.8%) |  | 1 (1.9%) | 4 (7.5%) |  |
|  | Others^¥^ | 32 (41.6%) | 11 (14.3%) |  | 14 (18.2%) | 2 (2.6%) |  | 12 (15.6%) | 4 (5.2%) |  |
| Occupation | Unemployed | 11 (42.3%) | 3 (11.5%) | > 0.05 | 7 (26.9%) | 0 (0%) | > 0.05 | 3 (11.5%) | 4 (15.4%) | 0.054 |
|  | Private employed | 4 (12.5%) | 2 (6.2 %) |  | 3 (9.4%) | 2 (6.2%) |  | 0 (0%) | 5 (15.4%) |  |
|  | Government employed | 12 (30%) | 6 (15%) |  | 1 (2.5%) | 2 (5%) |  | 1 (2.5%) | 2 (5%) |  |
|  | Self employed | 5 (20%) | 3 (12 %) |  | 3 (12%) | 0 (0%) |  | 3 (12%) | 0 (0%) |  |
|  | Others^£^ | 12 (21.1%) | 7 (12.3%) |  | 8 (14%) | 0 (0%) |  | 4 (7%) | 4 (7%) |  |
|  | Not applicable | 28 (38.9%) | 10 (13.9%) |  | 14 (19.4%) | 3 (4.2%) |  | 12 (16.7%) | 5 (6.9%) |  |
| Family income (monthly in birr) | <5000 | 49 (27.7%) | 20 (11.3%) | > 0.05 | 26 (14.7%) | 2 (1.1%) | 0.048 | 16 (9%) | 12 (6.8%) | > 0.05 |
|  | 5000-10000 | 16 (25.8%) | 10 (16.1%) |  | 7 (11.3%) | 5 (8.1%) |  | 6 (9.7%) | 6 (9.7%) |  |
|  | >10000 | 7 (53.8%) | 1 (7.7%) |  | 3 (23.1%) | 0 (0%) |  | 1 (7.7%) | 2 (15.4%) |  |
| Duration of illness | < 7 days | 56 (28.7%) | 24 (12.3%) | > 0.05 | 29 (14.9%) | 5 (2.6%) | > 0.05 | 20 (10.3%) | 14 (7.2%) | > 0.05 |
|  | 7-28 days | 12 (31.6%) | 4 (10.5%) |  | 5 (13.2%) | 0 (0%) |  | 2 (5.3%) | 3 (7.9%) |  |
|  | > 28 days | 4 (21.1%) | 3 (15.8%) |  | 2 (10.5%) | 2 (10.5%) |  | 1 (5.3%) | 3 (15.8%) |  |
| Hospitalization history | Yes | 25 (41%) | 7 (11.55) | 0.045 | 14 (23%) | 0 0(%) | 0.033 | 10 (16.4%) | 4 (6.6%) | > 0.05 |
|  | No | 47 (24.6%) | 24 (12.6%) |  | 22 (11.5%) | 7 (3.7%) |  | 13 (6.8%) | 16 (8.4%) |  |
| Surgery | Yes | 11 (22.9%) | 7 (14.6%) | > 0.05 | 5 (10.4%) | 1 (2.1%) | > 0.05 | 3 (6.2%) | 3 (6.2%) | > 0.05 |
|  | No | 61 (29.9%) | 24 (11.8%) |  | 31 (15.2%) | 6 (2.9%0 |  | 20 (9.8%) | 17 (8.3%) |  |
| Comorbidity | Yes | 21 (27.3%) | 9 (11.7%) | > 0.05 | 13 (16.9%) | 2 (2.6%) | > 0.05 | 7 (9.1%) | 8 (10.4%) | > 0.05 |
|  | No | 51 (29.1%) | 22 (12.6%) |  | 23 (13.1%) | 5 (2.9%) |  | 16 (9.1%) | 12 (6.9%) |  |
| Antibiotic exposure in the last 90 days | Yes | 35 (40.7%) | 10 (11.6%) | 0.008 | 18 (20.9%) | 0 (0%) | 0.019 | 14 (16.3%) | 4 (4.7%) | 0.009 |
|  | No | 37 (22.3%) | 21 (12.7%) |  | 18 (10.8%) | 7 (4.2%) |  | 9 (5.4%) | 16 (9.6%) |  |

**Note:** ¥ Other Include study participants not eligible to education (less than 7 years); £ other Include retired, farming, doing paid work, and non-regular businesses.
